# Hybrid Nanoparticles and Composite Hydrogel Systems for Delivery of Peptide Antibiotics

**DOI:** 10.3390/ijms23052771

**Published:** 2022-03-02

**Authors:** Dmitrii Iudin, Marina Vasilieva, Elena Knyazeva, Viktor Korzhikov-Vlakh, Elena Demyanova, Antonina Lavrentieva, Yury Skorik, Evgenia Korzhikova-Vlakh

**Affiliations:** 1 Institute of Macromolecular Compounds, Russian Academy of Sciences, Bolshoi VO 31, 199004 St. Petersburg, Russia; dmitriy-yudin97@mail.ru (D.I.); mazarine@list.ru (M.V.); yury_skorik@mail.ru (Y.S.); 2 Institute of Chemistry, St. Petersburg State University, Universitetskii 26, Peterhof, 198504 St. Petersburg, Russia; v.korzhikov-vlakh@spbu.ru; 3 State Research Institute of Highly Pure Biopreparations, Pudozhsakya 7, 197110 St. Petersburg, Russia; e@knyazeva32.ru (E.K.); lenna_22@mail.ru (E.D.); 4 Institute of Technical Chemistry, Gottfried-Wilhelm-Leibniz University of Hannover, 30167 Hannover, Germany; lavrentieva@iftc.uni-hannover.de

**Keywords:** hybrid nanoparticles, core–shell structures, peptides, polymyxin, colistin, drug delivery systems, antibiotics, antimicrobial properties, composite materials

## Abstract

The growing number of drug-resistant pathogenic bacteria poses a global threat to human health. For this reason, the search for ways to enhance the antibacterial activity of existing antibiotics is now an urgent medical task. The aim of this study was to develop novel delivery systems for polymyxins to improve their antimicrobial properties against various infections. For this, hybrid core–shell nanoparticles, consisting of silver core and a poly(glutamic acid) shell capable of polymyxin binding, were developed and carefully investigated. Characterization of the hybrid nanoparticles revealed a hydrodynamic diameter of approximately 100 nm and a negative electrokinetic potential. The nanoparticles demonstrated a lack of cytotoxicity, a low uptake by macrophages, and their own antimicrobial activity. Drug loading and loading efficacy were determined for both polymyxin B and E, and the maximal loaded value with an appropriate size of the delivery systems was 450 µg/mg of nanoparticles. Composite materials based on agarose hydrogel were prepared, containing both the loaded hybrid systems and free antibiotics. The features of polymyxin release from the hybrid nanoparticles and the composite materials were studied, and the mechanisms of release were analyzed using different theoretical models. The antibacterial activity against *Pseudomonas aeruginosa* was evaluated for both the polymyxin hybrid and the composite delivery systems. All tested samples inhibited bacterial growth. The minimal inhibitory concentrations of the polymyxin B hybrid delivery system demonstrated a synergistic effect when compared with either the antibiotic or the silver nanoparticles alone.

## 1. Introduction

The emergence of antibiotics revolutionized the treatment of infectious diseases and made a significant contribution to reducing the associated morbidity and mortality. However, the year-by-year growth in the use of antibiotics has led to the appearance of bacterial resistance to these drugs. Today, the increasing number of drug-resistant pathogenic bacteria poses a global threat to human health. Despite the fact that new antibiotics are actively being researched to overcome the resistance of microorganisms to antibacterial drugs, a steady and gradual reduction in the introduction of new drugs has been reported [1].

**Figure 1:**
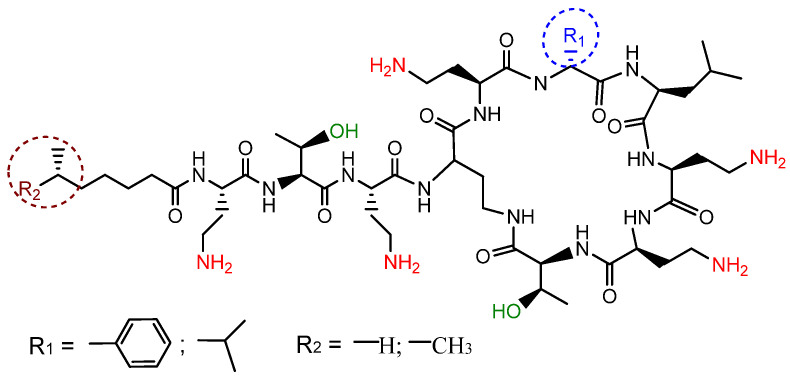
General structure of polymyxins.

In the past decade, failures in the treatment of multidrug-resistant bacterial infections have led to the return of some antibiotics previously rejected by medical practice. These include the polymyxins (PMXs), a group of cyclic peptide antibiotics consisting of encoded and non-encoded amino acids (Figure 1) and active mainly against Gram-negative pathogens [2]. Polymyxin B contains phenylalanine in the peptide structure, which is replaced by leucine in polymyxin E. Furthermore, both polymyxin B and E exist as mixtures of closely related lipopeptides differing in the structure of the *N*-terminal fatty acyl group, namely, 6-methyloctanoic acid (form B1 or E1) or 6-methylheptanoic acid (form B2 or E2). This group of antibiotics was introduced into clinical practice more than 50 years ago, but they were quickly discovered to have serious side effects, such as nephro- and neurotoxicity [3]. However, in 2011, the World Health Organization reclassified PMXs as extremely important antibiotics in practical health care for the treatment of infections for which there are now practically no alternatives [4].

The return of PMXs as therapeutic agents in clinical practice has prompted a search for ways to minimize their side effects. The emergence of nanomaterials has presented one strategy for developing drug delivery systems that can optimize the treatment of infectious diseases while minimizing the toxic effects of drugs like PMXs [5,6,7]. PMXs are positively charged, so they can interact with anionic phospholipids and anionic polymers; therefore, the modern delivery systems considered for PMXs include anionic liposomes [8,9,10] and anionic polymers. The anionic polymers form polyelectrolyte complexes (PECs) with PMXs due to electrostatic interactions [11,12]. For instance, Liu et al. reported the preparation of nanoparticles (NPs) by complexation of colistin (polymyxin E) with poly(glutamic acid) and further stabilization of this delivery system using 1,2-dimyristoyl-sn-glycero-3-phosphoethanolamine-N-(methoxy (polyethylene glycol)-2000) [11]. Dubashynskaya et al. evaluated the efficiency of hyaluronan/colistin PECs as PMX delivery systems [12]. The positively charged PMXs can also be successfully loaded into PECs formed by combinations of polycations and polyanions. For example, Coppi et al. developed pH-sensitive alginate/chitosan PECs for oral administration of PMX B [13,14]. Similar systems based on PECs formed by a polycation (oligochitosan) and a polyanion (anionic starch) for co-delivery of colistin and tobramycin were recently studied by Yasar et al. [15]. Hyaluronan/diethylaminoethyl chitosan PECs for improved colistin delivery have been reported by Dubashynskaya et al. [16].

The PMX structure, which includes a lipophilic tail and a hydrophobic amino acid (phenylalanine or leucine), allows PMX loading into lipid NPs and polymers, as well as into amphiphilic copolymers. Recently developed PMX delivery systems have included not only lipid NPs [17,18] and hydrophobic poly(butyl cyanoacrylate)-based NPs [19], but also poly(L-lactide)/halloysite nanotube nanofiber mats for wound treatment [20]. Therefore, in addition to systemic administration (e.g., parenteral administration), PMXs can also be used for external treatment to prevent skin infections in cases of postsurgical or burn wounds [21]. The conventional forms for prolonged external skin treatments with PMXs include patches [22] and hydrogel films [23,24]. Innovative microneedle technologies can also be a choice for effective transdermal delivery of these antibiotics [25,26]. Compared to systemic administration, local delivery of antibiotics has several advantages, including reduced systemic toxicity, high therapeutic efficacy, and low occurrence of bacterial resistance. Several biopolymers, such as hyaluronic acid, sodium alginate, chitosan, collagen, and dextran, have been widely used to prepare materials for prolonged and safe antibacterial wound treatment [24,27,28].

Like many other antibiotics PMXs can be used together with silver NPs (Ag NPs), which already have their own antimicrobial activity against different microorganisms, including *Staphylococcus aureus*, *Vibrio cholerae*, *Pseudomonas aeruginosa*, *Bacillus subtilis*, *Escherichia coli*, etc. [29,30]. One of the proposed mechanisms of the antimicrobial activity of Ag NPs is associated with a gradual release of silver ions (Ag^+^), which actively interact with the cell membrane and penetrate into the microorganism. The internalized Ag^+^ ions deactivate the respiratory enzymes, thereby blocking adenosine triphosphate production but retaining electron transport, which then supports the generation of reactive oxygen species that can modify DNA. The Ag^+^ ions can also bind to the phosphate groups of DNA, thereby interfering with DNA replication [30]. Brown et al. compared the biological activity of Ag and Au NPs, as well as the same NPs functionalized with the beta-lactam antibiotic ampicillin [31]. The authors found no antibacterial effect with neat Au NPs, whereas the NPs modified with the antibiotic showed antimicrobial activity. By contrast, the neat Ag NPs clearly had antibacterial activity against Gram-positive and Gram-negative bacteria, even without the presence of ampicillin. Manukumar et al. reported that a preparation of Ag NPs coated with chitosan and capable of incorporating thymol showed broad bactericidal activity against both Gram-positive and Gram-negative bacteria [32]. Moreover, in some studies, antibiotics loaded into hybrid polymer–silver NPs have demonstrated enhanced antibacterial activity [33,34].

Earlier, we studied the self-assembly of amphiphilic poly(glutamic acid-*co*-polyphenylalanine) into NPs of ca. 200 nm and the use of these NPs as delivery systems for both PMXs B and E [35,36]. The developed NPs did not show cytotoxicity but demonstrated reduced uptake by macrophages. PMX B, being slightly more hydrophobic than PMX E, demonstrated a higher loading and a stronger retention than PMX E. The minimal inhibitory concentrations for the developed formulations were equal to those of the free antibiotics.

In the present study, we prepared hybrid core–shell NPs consisting of an Ag core covered by a poly(glutamic acid) (PGlu) shell, and we investigated these PGlu@Ag NPs as delivery systems for PMXs B and E. The Ag core was chosen to enhance the antibacterial effect of the PMX formulation, while PGlu was selected because of its effective PMX binding and the absence of cytotoxicity. The hybrid PMX/PGlu@Ag NPs were carefully characterized in terms of their size, polydispersity, and morphology. The loading of PMXs was evaluated to optimize the amount of loaded drug and the stability of the formulation. The PMX/PGlu@Ag NPs were used further as a filler to prepare agarose-based hydrogel composites that could be considered as systems for antibacterial treatment of skin damage. The release kinetics of PMX from both hybrid and composite delivery systems were investigated and analyzed using different mathematical models to predict the most probable mechanisms of release. The antimicrobial efficacy against *P. aeruginosa* was evaluated for both types of PMX delivery systems developed in this study.

## 2. Results and Discussion

### 2.1. Synthesis of SH-PGlu

The preparation of PGlu@Ag NPs was based on the reduction of Ag^+^ in silver nitrate to Ag^0^ by sodium borohydride in the presence of thiol-containing PGlu as a coating and stabilizing agent.

**Figure 2:**
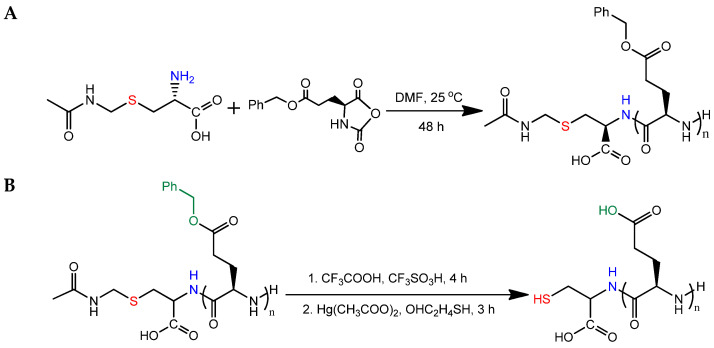
Scheme of synthesis of PGlu containing a terminal SH-group: (**A**) ROP and (**B**) deprotection.

PGlu containing a terminal thiol group was obtained by the ring-opening polymerization of N-carboxyanhydride (NCA) of L-glutamic acid γ-benzyl ester (Glu(OBzl)) using S-acetamidomethyl-L-cysteine (Cys(Acm)) as an initiator (its amino group can initiate ring opening polymerization (ROP) via the amine mechanism). The scheme of the synthesis is illustrated in Figure 2A. At this step, the polymer had a hydrophobic nature. According to size-exclusion chromatography (SEC), the synthesized protected polymer had the following molecular weight characteristics: *M_w_* = 5700, M_n_ = 4300, Đ = 1.32.

In the next step, the obtained polymer was deprotected (Figure 2B). Initially, the γ-carboxyl groups of glutamic acid were deprotected using trifluoracetic/trifluoromethanesulfonic acid (TFA/TFMSA) solution. Finally, the Acm protection of the terminal cysteine was removed to generate a free thiol group. The presence of the SH-group in PGlu was testified by Ellman’s test [37]. The product of the reaction with Ellman’s reagent is easily detected spectrophotometrically at a wavelength of 412 nm and can be quantified. The content of thiol groups in the polymer was 2.28 mg, which corresponded to 70 ± 5 wt% of the thiol groups in S-acetamidomethyl-L-cysteine taken as the initiator of the polymerization reaction.

### 2.2. Preparation of Hybrid Nanoparticles

**Figure 3:**
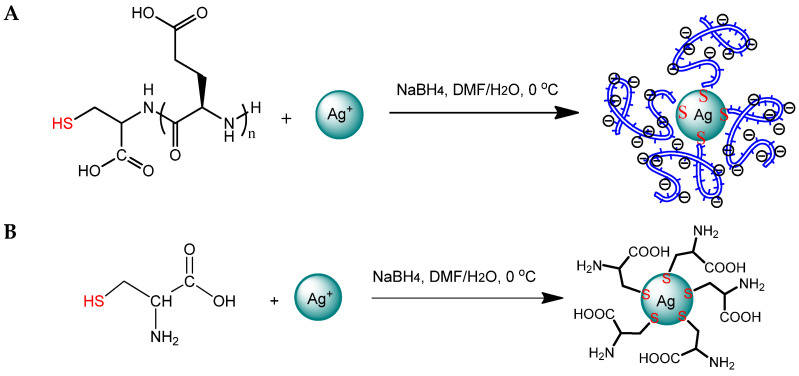
Scheme of preparation of PGlu@Ag (**A**) and Cys@Ag nanoparticles (**B**).

Hybrid NPs were obtained via the reduction of the Ag^+^ in silver nitrate with sodium borohydride with a simultaneous stabilization of the generated NPs by SH-PGlu due to the formation of -S-Ag bonds. The scheme of the reaction is shown in Figure 3A. During the reaction, the suspension changed in color from light beige to a rich golden brown, indicating that silver ions were reduced, and that stabilized NPs had formed. Ag NPs stabilized with a low molecular weight compound, cysteine (Cys@Ag), were also obtained for comparison (Figure 3B).

The synthesized NPs were analyzed by dynamic and electrophoretic light scattering (DLS and ELS) methods in various media to determine hydrodynamic diameter (D_H_), polydispersity index (PDI), and ζ-potential. Table 1 shows that the hybrid NPs had hydrodynamic diameters close to 90–100 nm in all studied media. As expected, the PGlu@Ag NPs demonstrated a strong negative charge and provided high stability to the colloid system.

**Figure 4:**
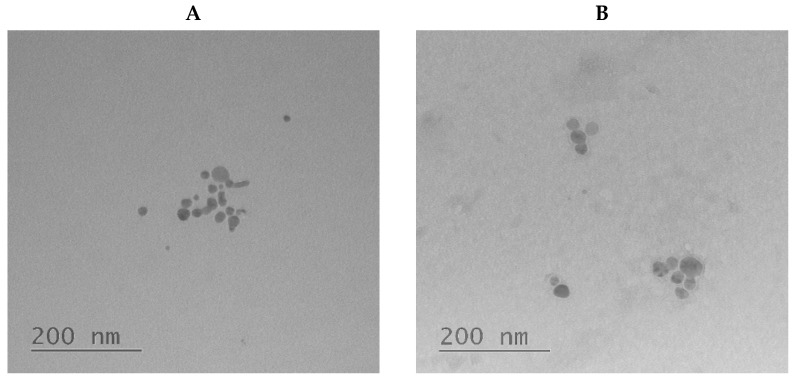
TEM images of PGlu@Ag nanoparticles without staining (**A**) and with uranyl acetate staining (**B**).

The morphology of the synthesized hybrid particles was investigated by TEM (Figure 4) using non-contrasted NPs and NPs stained with uranyl acetate. In the first case, the detection of only the silver core is possible, while the use of uranyl acetate allowed the detection of the size of the particle complete with its polymer shell.

The DLS results for Cys@Ag NPs revealed that they were considerably larger than PGlu@Ag NPs (Table 2). Most likely, this result could be associated with less stabilization of the reducing Ag by cysteine. The Cys@Ag NPs have both amino- and carboxylic groups on their surface; therefore, they showed the ability to change the ζ-potential depending on the medium in which they were redispersed.

In addition, PGlu@Ag and Cys@Ag NPs obtained in PBS were studied by nanoparticle tracking analysis (NTA) (Figure S1 of Supplementary Materials). The hydrodynamic diameter of NPs determined by NTA is in agreement with the average D_H_ obtained by DLS: Ag NPs stabilized by PGlu had smaller size than those stabilized by small molecule Cys. In both cases, DLS and NTA indicate fairly wide size distribution of NPs, but, unlike PGlu@Ag NPs, a multimodal distribution was detected for Cys@Ag NPs. The appearance of several modes for Cys@Ag may be related to the poor stabilization of Ag by a small ligand as well as to the aggregation of particles due to the electrostatic interaction between the amino and carboxyl groups that are present in each cysteine molecule and ionized at pH 7.4.

### 2.3. Biological Evaluation of Hybrid Nanoparticles

**Figure 5:**
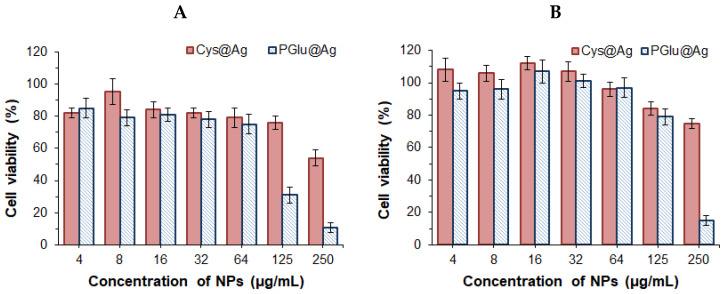
Viability of HEK 293 (**A**) and HepG2 (**B**) incubated with hybrid nanoparticles for 72 h.

The cytotoxicity of the hybrid NPs was evaluated using HEK 293 and HepG2 cell lines by the CTB test after 72 h (Figure 5).

In the case of Cys@Ag NPs, the IC_50_ values for normal (HEK 293) and cancer (HepG2) cells were 284.4 ± 9.8 and 342.1 ± 16.7 µg/mL, respectively. In turn, IC_50_ values for PGlu@Ag NPs were 84.9 ± 6.3 and 192.8 ± 9.5 µg/mL for HEK 293 and HepG2, respectively. Taking into account that PGlu is a non-toxic polymer (IC_50_ > 1000 μg/mL) [36,38], the cytotoxic effect of PGlu@Ag NPs at concentrations higher than 64 μg/mL is evidently provided by the silver core of the NPs. According to the published data, Ag NPs are quite toxic to mammalian cells. The reported cytotoxicity of non-coated Ag NPs with a size of 20–50 nm is in the range of 5–15 μg/mL for normal cells and 40 μg/mL for the cancerous cells (MCF-7) [39,40]. In turn, coating of the Ag NPs with a low molecular or polymer shell has been shown to improve their compatibility with cells [40,41]. In particular, the Janus PEG@Ag NPs 40 nm in size appeared to be non-toxic to HepG2 cells at concentrations up to 64 μg/mL [42]. Tang et al. reported IC_50_ values for Fe_3_O_4_@*T. spicata*/Ag NPs with a size of 50 nm of 289, 311, and 174 µg/mL against Ramos.2G6.4C10, HCT-8 [HRT-18], and HCT 116 cancer cell lines, respectively [43], while normal cells (HUVECs) retained their viability in the presence of up to 1000 μg/mL Fe_3_O_4_@*T. spicata*/Ag. The cytotoxicity of Ag NPs is associated with their size; smaller Ag NPs demonstrate higher cytotoxicity, presumably due to their faster penetration into the cells [40]. This may be the reason for the higher cytotoxicity of PGlu@Ag (~100 nm) compared to CyS@Ag (~420 nm) in HEK 293 and HepG2 cells.

**Figure 6:**
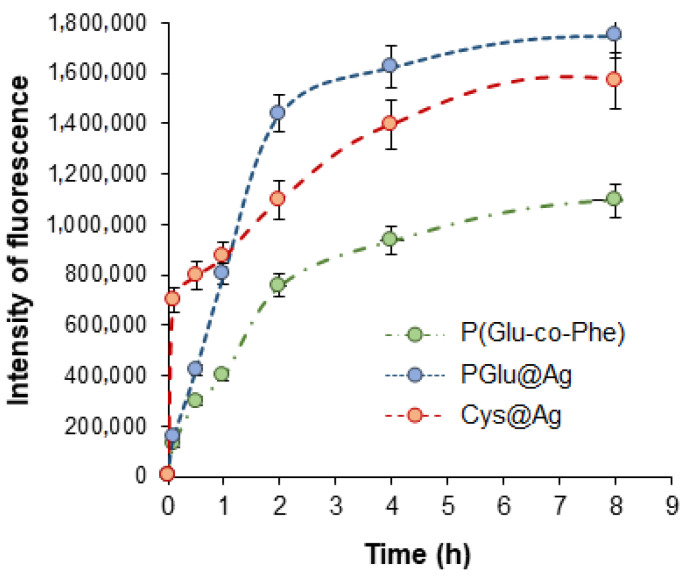
Uptake of different nanoparticles by macrophages (J7741.A).

Flow cytometry was used to study the uptake of NPs by macrophages. Besides the two hybrid NP types, the poly(L-glutamic acid-co-L-phenylalanine) (P(Glu-co-Phe)) NPs developed earlier were also applied as a benchmark. We have recently shown that uptake of P(Glu-co-Phe) about 200 nm in size by macrophages was reduced in comparison with widely used PEG-*b*-PLA NPs [36]. The capture study was carried out by incubation of mouse macrophages (J774.1A) with Cy5-labeled NPs for 0–8 h. As shown in Figure 6, the lowest uptake by macrophages was observed for the control P(Glu-co-Phe) NPs. Both kinds of Ag-based NPs demonstrated higher uptake by macrophages than was observed for the polymer NPs. Several factors, such as shape, size, surface charge, and rigidity, are known to affect the rate of phagocytosis [44,45]. Taking into account that all the tested NPs are spherical and negatively charged under the conditions of the experiment, the most probable reason for such behavior of the Ag-based NPs is their higher rigidity in comparison to the self-assembled P(Glu-co-Phe) NPs. Indeed, the published data clearly indicate that softer NPs demonstrate reduced uptake efficiency, which is associated with their tendency to undergo local deformation upon contact with macrophages [44]. Compared to PGlu@Ag, the silver NPs stabilized with cysteine (Ag@Cys) demonstrated an enhanced uptake during the first hour, while the total uptake after 8 h was slightly lower. This trend might be explained by the influence of the rigidity factor at the initial step and the size factor at a subsequent step. In particular, a larger sized NP is known to require more time for uptake, as the cell needs to build a larger phagocytic cup [44]. In general, the rate of uptake detected for PGlu@Ag is comparable to that established by us earlier for rigid PEG-b-PLA NPs of a similar size (~100 nm) [36]. Thus, it can be concluded that the coating of silver NPs with PGlu demonstrated a satisfactory result and that the hybrid NPs can be considered as viable delivery systems.

**Figure 7:**
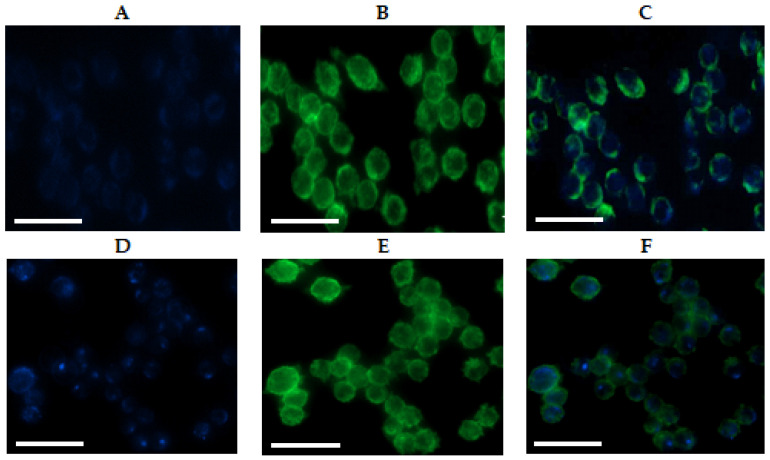
Fluorescence images of J7741.A cells treated for 3 h with: (**A**–**C**) hybrid PGlu@Ag nanoparticles labeled with Cy5; (**D**–**F**) Cy5-P(Glu-co-Phe) nanoparticles. The images from left to right show the fluorescence of Cy5-labeled nanoparticles in cells (blue), the stained cell membrane (green), and the overlap of two images (scale bar equal to 50 μm).

As an additional experiment, the uptake of NPs by macrophages was visualized by fluorescence microscopy (Figure 7) after 3 h of co-incubation. Analysis of the images supported the results of flow cytometry regarding the capture of NPs by macrophages by nonspecific endocytosis.

### 2.4. Preparation of Polymyxin Formulations Based on Hybrid Nanoparticles

**Figure 8:**
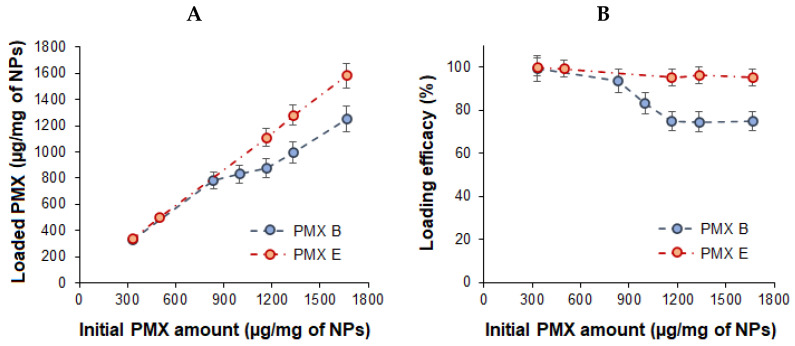
Drug loading (DL) (**A**) and loading efficacy (LE) (**B**) for polymyxins B and E loaded into hybrid nanoparticles.

PMXs were loaded by exploiting the polyelectrolyte interactions between the positively charged PMX B or E and the negatively charged PGlu. Drug loading (DL) and loading efficacy (LE) are important parameters of drug delivery systems. The dependence on DL and LE obtained for loading of PMXs B and E into PGlu@Ag is shown in Figure 8. Both antibiotics demonstrated high binding ability to PGlu, in agreement with the results previously observed for P(Glu-co-Phe) [36]. The loading of more than 1000 µg of PMX per mg of NPs occurred to be possible for both antibiotics. However, the LE was slightly higher for the more hydrophilic PMX E than for PMX B.

Monitoring of the hydrodynamic diameter and ζ-potential of the delivery systems revealed a considerable change in these characteristics with an increase in PMX loading (Table 3). The loading up to 450 µg/mg of NPs ensured the formation of stable compositions with *D_H_* up to 210 nm. A further increase in the PMX loading led to a sharp increase in the hydrodynamic diameter (more than 1 µm) with simultaneous reduction in ζ-potential that, in turn, followed by the instability of the formulation over time. Thus, the loading of more than 450 µg of PMX per mg of NPs seems impractical.

### 2.5. Composite Delivery Systems

As mentioned earlier, the composite materials based on hydrogels may have potential applications as systems for wound treatment. In this work, agarose was selected to prepare the composite hydrogels for use as wound coatings. Agarose is an inexpensive natural hydrophilic polymer obtained from various algal species. The key property of agarose is its ability to form hydrogels due to the formation of hydrogen bonds between saccharide units at room temperature. Agarose-based hydrogels are biocompatible and non-toxic, making them promising for applications in medicine.

**Figure 9:**
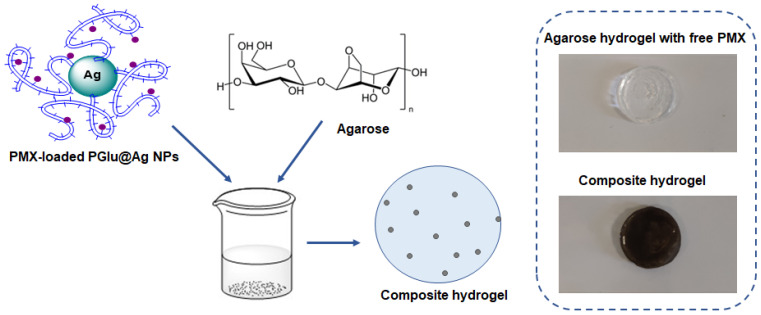
Scheme for the preparation of composite hydrogels with antimicrobial properties. The concentration of hybrid nanoparticles in the composite gel shown was 5 mg per specimen (0.3 mL of hydrogel).

Composite films were manufactured by casting a warm agarose solution containing free antibiotics or hybrid PMX-loaded PGlu@Ag NPs onto the surface of a plastic substrate and then cooling to room temperature. The scheme for obtaining the composite material and images of hydrogels loaded with free PMX B and hybrid delivery systems are illustrated in Figure 9. The loading was 1.0–2.5 mg PMXs per 0.3 mL of hydrogel.

### 2.6. Release of Polymyxins from Hybrid and Composite Systems

**Figure 10:**
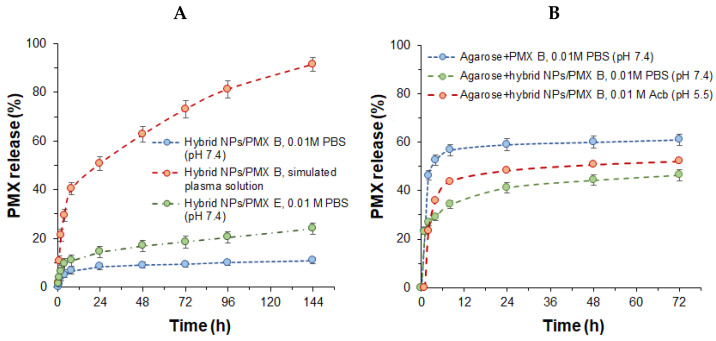
Release of polymyxin E and B over time from hybrid PGlu@Ag nanoparticles (**A**) and polymyxin B from composite hydrogels (**B**).

As was noted by several groups, the greater hydrophilicity of PMX E leads to less retention in formulations than is seen with PMX B; therefore, PMX E shows a faster release [8,9]. In the present study, the cumulative release of PMX E from PGlu@Ag NPs in 0.01 M PBS (pH 7.4) was two times faster than for PMX B (Figure 10A). These data are in agreement with our previous study, in which the release of both PMX E and B was studied from PGlu-containing NPs [36]. Moreover, in that study, we observed a considerable release of PMXs into human blood plasma. Here, to compare the effect of the presence of competitive macromolecules, the release of PMX B into a simulated plasma solution at 37 °C was monitored for 6 days (Figure 10A). As expected, the presence of competing protein macromolecules (human serum albumin, HSA) in solution caused a pronounced release, which reached almost 90% over the tested period.

In the case of composite materials, the rate of release of PMX B was studied in 0.01 M PBS (pH 7.4) and compared with the release of a free antibiotic from the hydrogel (Figure 10B). A release of 60% of the PMX B was observed within 6 h for the antibiotic-loaded hydrogel, whereas the release was only half that value for the composite material for the same time. A comparable rate of PMX B release has also been reported recently by Shi at al., who investigated hydrogels from self-assembling peptide amphiphiles containing negatively charged carboxyl groups and loaded with PMX B [46]. In particular, the release of 60–80% PMX B after 60 h was documented by those authors.

The release from composite materials in acidic buffer (0.01 M acetic buffer, pH 5.5) was more pronounced than for the same material in 0.01 M PBS (pH 7.4) (Figure 10B). This result can be explained by better ionization of PMX amino groups in acidic buffer and, as a result, faster diffusion of peptide into the medium.

**Figure 11:**
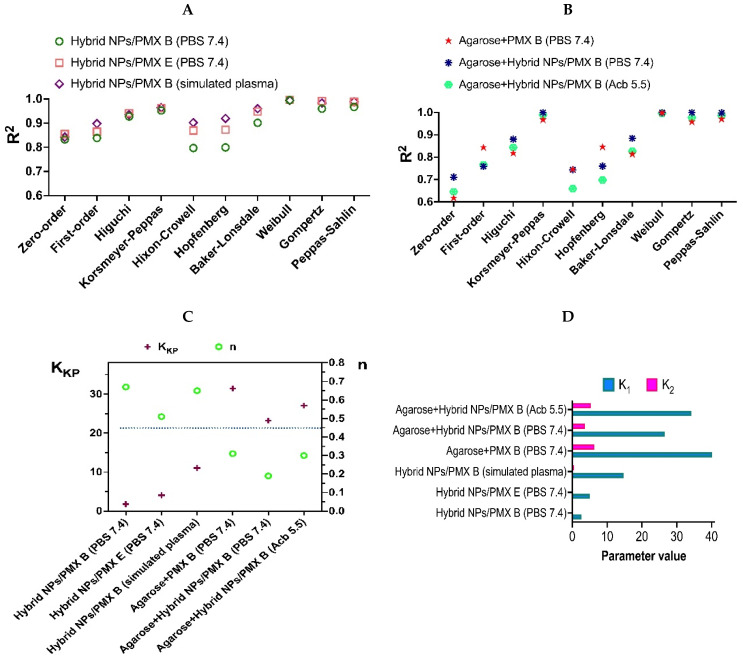
Results of mathematical analysis of PMX release profiles with application of standard models: (**A**) comparison of correlation coefficients of the regressions obtained with different models for release from hybrid NPs; (**B**) comparison of correlation coefficients of the regressions obtained with different models for release from agarose gel and composite gels; (**C**) results obtained by application of the Korsmeyer–Peppas model, *K_KP_*—release rate constant from the Korsmeyer–Peppas equation, *n*—parameter from Korsmeyer–Peppas equation showing the mechanism of drug release; (**D**) results obtained by application of Korsmeyer–Peppas model, *K*_1_—impact of diffusional mechanism, *K*_2_—impact of relaxation on release.

The obtained PMXs release profiles were analyzed by fitting them with several mathematical models to elucidate the mechanisms of peptide release. All regression curves and calculated data are presented in the Supplementary Materials (Table S1 and Figure S2). Here we present some conclusions from the analysis of these data [47]. First, it is obvious that release of polymyxins from both hybrid NPs and gels could hardly be fitted with zero-order and first-order models (Figure 11A,B). Thus, the release of peptides is not only a function of time, and the rate of the process is proportional not only to the amount of remaining drug in the matrix. The release process is more complex because it involves peptide–polymer interactions. It is also evident that polymyxins’ release profiles poorly correlate with Hixson–Crowel and Hopfenberg models. This means that dissolutions and matrix erosion could not be considered as rate-limiting processes for release. Among standard models the best fitting was observed for Higuchi and Baker-Lonsdale models, revealing the fact that diffusion is the most important process for peptides’ release from the systems under study.

Quite good correlation was obtained with Korsmeyer–Peppas model by using initial stage of release. The correlation coefficients allow to consider and analyze the rate constant (*K_KP_*) and *n* parameters for different systems under investigation (Figure 11C). One can observe that for hybrid NPs the *n* parameter is above 0.45 (above the dividing line on Figure 11C), which means that mechanism of both PMX B and PMX E release is controlled by non-Fickian diffusion. In the case of gels, the *n* is below the dividing line, so the mechanism of release corresponds to Fickian diffusion. The release rate constants were lower for all hybrid NPs, than those for released from the agarose gel and composite gels.

The obtained results also show very good correlation with the Weibull, Gompertz, and Peppas–Sahlin models. The first model allows us to conclude that the release has some latency time (Ti) and could be described by parabolic curve (β < 1) [47]. Good correlation with the Gompertz model looks quite rational because this model describes the release of drugs possessing good solubility and an intermediate release rate [48]. The best results with this model were obtained with PMX E release from hybrid NPs (R^2^ = 0.9915) and for PMX B release from composite agarose gel loaded with hybrid NPs (R^2^ = 0.9993). Good fitting of the full release curves with the Peppas–Sahlin model allowed us to evaluate the impact of diffusion and relaxation on the mechanism of peptides release (Figure 11D). The values of constants from this model, namely *K*_1_ and *K*_2_, show the effect of diffusion and relaxation, correspondingly, on the process of release. One can observe that diffusion is the major factor affecting peptide release for all systems under study. The polymer matrix relaxation effect in the case of hybrid NPs is negligible. However, such relaxation seems to be important in the case of systems containing agarose. This looks quite rational because peptides need to diffuse through gel layer in those cases.

Thus, we can conclude that diffusion is the main mechanism of peptide release from the systems under study. The nature of PMX, the release medium and the gel layer affect the rate of release, but not the mechanism. The release is faster in simulated plasma (in the case of hybrid NPs) and in acetate buffered solution, pH 5.5, than in phosphate buffered solution, pH 7.4, but the diffusion control of the release is acting in all these systems.

### 2.7. Antimicrobial Activity

The antimicrobial activity of the PMX hybrid formulations was evaluated by determining the MIC against *P. aeruginosa*. The PGlu@Ag NPs loaded with PMX E or B effectively suppressed the growth of *P. aeruginosa*. For both formulations, the MIC was 1 µg/mL whereas the free PMX E and B demonstrated MICs of 1 and 4 µg/mL, respectively. For comparison, a previously developed delivery system based on P(Glu-co-Phe) NPs also demonstrated a MIC equal to 4 µg/mL for a system loaded with PMX B and 1 µg/mL for a system containing PMX E [36]. Thus, the higher antimicrobial properties of the PMX B formulation based on the hybrid NPs compared to a purely polymer delivery system or free antibiotic can be attributed to the synergistic effect of the antibiotic and the silver NPs. A synergistic effect of PMX B and Ag NPs has also been observed by Salman et al., who studied the inhibition of *P. aeruginosa* using PMX B solution and non-covered Ag NPs in a mixture [49].

**Figure 12:**
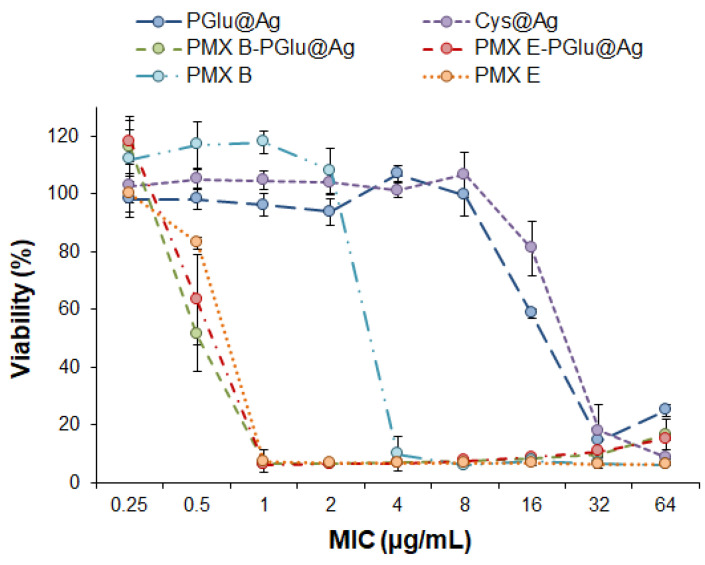
Antimicrobial activity of free polymyxins B and E, hybrid nanoparticles, and their nanoformulations against *Pseudomonas aeruginosa*. The data are given as mean ± SD (n = 3).

Both empty PGlu@Ag and Cys@Ag NPs demonstrated an evident inhibitory effect: a MIC of 32 µg/mL was determined for both silver-containing NPs (Figure 12). Since no difference was evident if the silver core was coated with a polymer or with a low molecular weight organic shell, the detected inhibitory activity may be related only to the presence of the silver core. Our previous evaluation of the inhibitory activity of P(Glu-co-Phe) NPs did not reveal any antimicrobial effect for PGlu-containing polymer NPs against *P. aeruginosa* [36]. Thus, even the presence of a polymer shell on the surface of silver NPs did not interfere with the manifestation of their antimicrobial activity.

**Figure 13:**
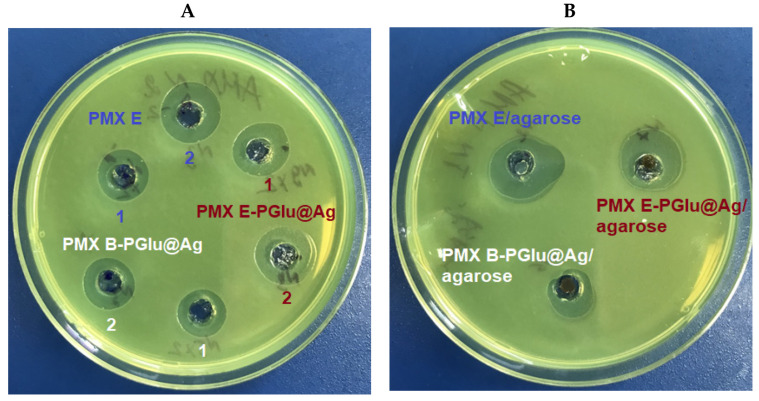
Suppression of *Pseudomonas aeruginosa* growth by exposure to different PMX formulations for 24 h: (**A**) dispersions of PMX hybrid delivery systems and free PMX as control: contents of the PMX in zones 1 and 2 were 37 ± 1 and 74 ± 2 µg/zone; (**B**) composite hydrogels and PMX in hydrogel as control (85.6 ± 1.7 µg of PMX per each zone).

The suppression of *P. aeruginosa* growth by composite materials was tested by the agar disk diffusion method. First, the suspensions of PMX B/E hybrid delivery systems were placed in wells formed in an agar plate and incubated for 24 h. In this case, the inhibitory activity directly depends on the rate of antibiotic release. Free PMX E was used as a control. Figure 13A shows that the inhibition zones due to free PMX E provided at two different amounts (37.3 ± 1.1 and 74.6 ± 2.2 µg/zone) were 14 (zone 1) or 16 mm (zone 2) in size. The inhibition zones produced by PMX E/PGlu@Ag formulations, taken at the same concentrations of PMX E, were 15 (zone 1) or 17 mm (zone 2) in size. In this case, the larger inhibition zones can be a result of both the fast release of PMX E from the hybrid systems and the antimicrobial activity of the silver NPs. At the same time, in the case of PMX B, which demonstrates a slower release rate than PMX E, the inhibition zones at the same concentrations of antibiotic were 13 (zone 1) and 14 mm (zone 2) in size.

A similar tendency was observed when testing the hydrogels. Composite hydrogels and a hydrogel containing PMX B as a control were loaded into the wells (85.6 ± 1.7 µg/zone) in the agar plate and incubated for 24 h. As in the previous case, the largest inhibition zone (19 mm) was detected for the hydrogel containing free PMX E (Figure 13B). As expected, the suppression of the bacterial growth provided by PMX E and B present in the composite hydrogels was proportional to the release rate of these antibiotics. In particular, the sizes of the inhibition zones for PMX E PGlu@Ag/agarose and PMX B PGlu@Ag/agarose were 17 and 13 mm, respectively.

Thus, all developed formulations demonstrated satisfactory antibacterial activity against *P. aeruginosa*. However, due to the faster release of PMX E, the inhibitory activity was higher for the PMX E formulations than for the PMX B formulations.

## 3. Materials and Methods

### 3.1. Chemicals and Supplements

L-glutamic acid-γ-benzyl ether (≥99.0%) (Glu(OBzl)), triphosgene (98%), α-pinene (98%), trifluoromethanesulfonic acid (TFMSA) (98%), S-acetamidomethyl-L-cysteine (Cys(Acm)) (99%), silver nitrate (≥99%), sodium tetaborate (99%), and human serum albumin (≥99%) were purchased from Sigma-Aldrich (Darmstadt, Germany) and used as received. Trifluoroacetic acid (≥99%) was purchased from Chemical Line LLC (St. Petersburg, Russia). Uranyl acetate was a product of Agar Scientific (Stansted, Essex, UK). Polymyxin B (sulfate) and polymyxin E (colistin sulfate) were acquired from Fluka (Munich, Germany) and BetaPharma (Wujiang, Shanghai, China), respectively. According to the previous HPLC-MS analysis of preparations, the commercial PMX B contained of 81.5% of PMX B1 and 18.5% of PMX B2 while the commercial PMX E contained 31.1% of PMX E1 and 68.9% of PMX E2 [36].

Dioxane, petroleum ether, ethyl acetate, and N,N-dimethylformamide (DMF) were purchased from Vecton (St. Petersburg, Russia), purified according to standard protocols, and dried before use.

Analytical-purity salts (Vecton, St. Petersburg, Russia) and deionized water were used to prepare buffer solutions. All buffer solutions were additionally filtered through a 0.45 µm Millex^®®^ membrane microfilter (Millipore Merck, Darmstadt, Germany). Spectra/Pore^®®^ (molecular weight cut-off (MWCO): 1000) dialysis bags were purchased from Spectra (Rancho Dominguez, CA, USA). Poly(methyl methacrylate) (PMMA) standards (*Mw* = 17,000−250,000; Ð ≤ 1.14) (Supelco, Bellefonte, PA, USA) were used for size exclusion chromatography (SEC) column calibration. The NPs self-assembled from poly(glutamic acid-*co*-phenylalanine) (P(Glu-co-Phe)) and used for comparisons in this work were prepared as described in our previous paper [36].

Human kidney embryonic cells (HEK 293), human liver carcinoma cells (HepG2), and mouse BALB/c monocyte macrophages (J774A.1) were acquired from Cell Lines Service GmbH (Eppelheim, Germany) and used for the evaluation of cytotoxicity and uptake of NPs by macrophages. A *P. aeruginosa* (ATCC 27,853 strain) culture was obtained from the collection of microorganisms of the State Research Institute of Highly Pure Biopreparations (St. Petersburg, Russia) and used to test the delivery systems for their minimum inhibitory concentrations (MICs).

### 3.2. Instruments

A VaCo 5-II Zirbus lyophilizer (Bad Grund, Germany) was used for freeze-drying. An ultrasonic homogenizer Sonopuls HD 2070 Bandelin Electronic (Berlin, Germany) was used for redispersion of the NPs. A Millipore Direct-Q 3 UV water purification system (Merck, Guyancourt, France) was used to purify water for a wide range of laboratory applications. A Zetasizer Nano ZS and Nanosight NS300, both from Malvern Instruments (Malvern, UK), were used for the determination of hydrodynamic diameter (*D_H_*), polydispersity index (*PDI*), and electrokinetic potential (ζ-potential) of the NPs. UV absorption measurements were carried out on a UV–1800 Shimadzu spectrophotometer (Kyoto, Japan).

All obtained copolymers were analyzed by SEC using a tandem of two Agilent PLgel MIXED-D columns (7.5 mm × 300 mm, 5 µm) (USA) and a Shimadzu LC-20 Prominence system supplied with a refractometric RID 10-A detector (Kyoto, Japan). The analysis was performed in a 0.1 M solution of LiBr in dimethyl formamide (DMF) as an eluent, at a flow rate of the mobile phase of 1 mL/min and a temperature of 40 °C. Molecular weight characteristics were calculated using GPC LC Solutions software (Shimadzu, Kyoto, Japan) and the calibration curve was plotted using PMMA standards.

NMR spectroscopy was carried out using a Bruker Avance III WB 400 MHz (Karlsruhe, Germany). A Shimadzu LC-20AD Prominence HPLC system (Kyoto, Japan) equipped with a mass spectrometric detector was used for PMX analysis.

A Jeol JEM-1400 transmission electron microscope with a maximum accelerating voltage of 120 kV was used to study the morphology of NPs.

A Thermo Fischer Fluoroscan Ascent FL fluorimeter (Bradenton, FL, USA) was utilized for the cytotoxicity study. A BD Accuri C6 flow cytometer (Becton Dickinson, Franklin Lakes, NJ, USA) was used to evaluate the cellular absorption of NPs. The visualization of fluorescently labeled cells was carried out with a Cytation 5 cell imaging multi-mode reader (Bad Friedrichshall, Germany).

### 3.3. Methods

#### 3.3.1. Synthesis and Characterization of Thiol-Containing PGlu

PGlu was obtained by the ring-opening polymerization (ROP) of L-glutamic acid-γ-benzyl ether N-carboxyanhydride (Glu(OBzl) NCA). Glu(OBzl) NCA was synthesized as described elsewhere [38,50] and polymerized in freshly distilled and anhydrous DMF. Cys(Acm) was used as an amine-type initiator (I). A Cys(Acm) solution in DMF was added to the monomer (M) solution in the same solvent to achieve a ratio of [M]/[I] = 35. The monomer concentration in the polymerization solution was 4 wt%. The polymerization was performed under stirring at 25 °C for 48 h. The obtained polymer was precipitated in 200 mL diethyl ether, and the precipitate was separated by centrifugation at 10,000 rpm for 7 min. The diethyl ether was decanted, and the precipitate was placed in a vacuum desiccator and dried for 24 h. The polymer yield was 79%. ^1^H NMR (DMSO-d_6_), δ (ppm): OBzl group: 7.33 (-C_6_H_5_) and 5.03 (-CH_2_); glutamic acid: 4.26 (-CH).

The obtained polymer was deprotected in two steps. First, the Bzl protective group was removed using a TFA/TFMSA solution. For this, 30 mg of polymer were dissolved in 1 mL trifluoroacetic acid and left in an ice bath with stirring for 30 min to completely dissolve the polymer. TFMSA (50 μL) was added, and the reaction medium was left for 3.5 h. The PGlu was then precipitated in diethyl ether, and the precipitate was separated by centrifugation at 10,000 rpm for 10 min. The resulting precipitate was dissolved in 5 mL DMF, and the solution was transferred to a dialysis bag (MWCO 1000). Dialysis was performed against deionized water for 48 h to remove low molecular weight impurities. The content of the dialysis bag was then freeze-dried. The yield of deprotected polymer was 85 wt%.

As the second step, the Acm-protective group was removed from the terminal cysteine, which had been used as an initiator in the polymerization process. The polymer was dissolved in 10 mL 30% acetic acid. A solution of mercury (II) acetate was added to the polymer solution at a ratio of 2 eq. of mercury (II) acetate per Acm-group of cysteine. The reaction was carried out for 1 h at room temperature with intensive stirring in an argon atmosphere. A 30% solution of a 10-fold excess of β-mercaptoethanol in 30% acetic acid was then added to the reaction mixture. The resulting medium was left for an additional 2 h with stirring under the same conditions. Finally, the reaction mixture was poured into a dialysis bag (MWCO 1000), and dialysis was performed for 8 h against deionized water with frequent replacement of the water. The content of the dialysis bag was then freeze-dried. The polymer yield after this step was 96 wt%.

The completeness of removal of the Acm-group was checked by performing an Ellman’s test to determine the presence of free thiol groups [51]. The test is based on the reaction of thiol with Ellman’s reagent, during which the disulfide bond of the reagent breaks, and yellow-colored 2-nitro-5-thiobenzoic acid is formed. In brief, polymer solutions (10 mg/mL) and Ellman’s reagent (4 mg/mL) were prepared in 0.05 M sodium phosphate buffer solution, pH 7.4, and 60 μL of Ellman’s reagent and 60 µL of the polymer solution were added to 3 mL of the working buffer and thoroughly mixed. After 20 min, the absorbance of the colored product was measured at 412 nm. The concentration of the thiol groups was calculated using the extinction coefficient (ε = 14,150 M^−1^ cm^−1^) [37].

#### 3.3.2. Preparation of Hybrid Nanoparticles

Aqueous solutions of 2.3 equivalents (eq.) of silver (I) nitrate and 6.3 eq. of sodium borohydride, calculated relative to the cysteine content in the polymer, were added to SH-containing PGlu dispersed in DMF at a concentration of 7 mg/mL. This method requires that silver nitrate be added first to the polymer solution at 0 °C and vigorously stirred, followed by dropwise addition of sodium borohydride solution, with stirring, over 25–30 min. During the reaction, a -S-Ag bond forms between the thiol group of the terminal cysteine and the reduced silver. The obtained NPs were centrifuged at 8000 rpm, then redispersed in 0.01 M sodium borate buffer, pH 9.3, and purified by dialysis against water for 48 h. The final dispersion of particles in water was frozen and freeze-dried.

Silver NPs stabilized with cysteine (Cys@Ag) were prepared in the same way as described above for PGlu@Ag. The only difference was that all components (cysteine, silver nitrate, and sodium borohydride) were initially dissolved in a small amount of water and then mixed in DMF. After sedimentation, the NPs were dispersed in 0.05 M MES (2-(N-morpholino)ethanesulfonic acid) buffer, pH 6.5, purified by dialysis against water for 48 h.

#### 3.3.3. Characterization of the Nanoparticles

The characteristics of the NPs, such as *D_H_*_,_ PDI and ζ-potential, were determined by dynamic and electrophoretic light scattering. Suspensions of NPs in different media with a volume of 1 mL and a concentration of 0.3 mg/mL were used for measurements.

Transmission electron microscopy (TEM) was used to study the morphology of the obtained hybrid NPs. Suspensions of NPs (0.3 mg/mL in water) were used for analysis. A drop (2–3 µL) of the colloids was placed on a copper grid (300 mesh) (Electron Microscopy Sciences, Hartfield, PA, USA) with the polymer (formvar) and carbon coatings and left in the air until completely dry. One part of the samples was additionally stained with 2 µL of a 2% uranyl acetate solution for 1 min, while the other part was left unstained. The grids with adhered samples at an accelerating voltage of 120 kV.

#### 3.3.4. Cytotoxicity and Uptake by Macrophages

The Hep G2 and HEK 293 cell lines were used to study the cytotoxicity of the NPs by placing 4 × 10^3^ cells into each well of a 96-well plate, followed by the addition of 200 µL DMEM-FBS medium containing basal medium, fetal bovine serum (FBS), penicillin, and streptomycin. The cells were cultured in a humidified atmosphere containing 5% CO_2_ at 37 °C for 24 h. The medium was then replaced with a fresh medium containing different concentrations of NPs (n = 3), and the plate was incubated at 37 °C for a further 72 h. The medium was removed, and the wells were filled with 10% CTB reagent solution in culture medium and incubated for 2 h. The CTB test is based on the ability of living cells to convert resazurin into the fluorescent product resorufin (λ_ex_. = 545 nm, λ_em_. = 590 nm), with the amount of conversion proportional to the number of viable cells [52]. The obtained data were expressed as a percentage of the control.

The J7741.A cell line (mouse macrophages) was used to study the uptake of NPs by macrophages by flow cytometry. Three types of samples fluorescently labeled with the same amount of Cy5 were prepared: Cys@Ag, PGlu@Ag, and P(Glu-co-Phe). For the experiment, 0.6 mL DMEM containing 15 × 10^4^ cells were placed into each well of a 24-well plate and cultivated in a humidified incubator with 5% CO_2_ at 37 °C for 24 h. The medium was then replaced with a fresh medium containing 0.02 mg/mL of the labeled NPs. The plates were incubated in 5% CO_2_ at 37 °C for 0–8 h, then the cells were detached with a cell lifter, centrifuged at 300 rpm for 5 min, and resuspended in 250 µL phosphate buffered saline (PBS; pH 7.4). The fluorescence signals were measured using a flow cytometer with a 488 nm argon-ion laser. Only viable cells (at least 30,000 events/sample) were used in the analysis.

The visualization of the uptake by macrophages was carried out in a 24-well black plate seeded with 15 × 10^3^ cells in 0.6 mL of culture medium. The cells were incubated with 0.1 µg/mL Cy5-labeled NPs in 5% CO_2_ at 37 °C for 3 h. The cell membranes were then stained with CellMask^TM^ green plasma membrane stain and visualized with a fluorescence microscope (20×).

#### 3.3.5. Loading of Polymyxins into Hybrid Nanoparticles

Hybrid NPs loaded with PMX were prepared using a 1 mg/mL antibiotic solution in 0.01 M sodium phosphate buffer (pH 7.4). Dried NPs in the same buffer were redispersed by ultrasonication for 20 s to prepare hybrid NPs (1 mg/mL). PMX solution (100–600 µL) was added to the 300 µL of the NP dispersion, and the solution volume was brought to 1 mL. The resulting mixture was thoroughly vortexed and left at 4 °C overnight. Other manipulations, as well as the protocol for PMX HPLC analysis, were as described in our previous paper [36].

#### 3.3.6. Preparation of Composite Materials

A weighed portion of agarose was placed into deionized water and heated to 90 °C to obtain a homogenous solution. Hybrid NPs loaded with PMX or free antibiotic were used as the fillers for the hydrogel matrix. To prepare a hydrogel, 100 µL of a PMX solution or the hybrid delivery system dispersion were placed into the wells of a 24-well plate, and 200 µL of the warm agarose solution was added, with mixing with a pipette tip. The final concentration of agarose was 1.5 wt%. The resulting mixture was rapidly cooled to form the hydrogel composites. The loading of PMX was varied from 1.0 to 2.5 mg per 0.3 mL of hydrogel. When the PMX hybrid delivery systems were used as fillers, the ratio of PMX to PGlu@Ag NPs was 1:2.

#### 3.3.7. Release of Polymyxins

The release of PMXs from hybrid NPs and composite materials was studied using 0.01 M PBS (pH 7.4) or simulated plasma solution (8% human serum albumin solution in 0.01 M PBS, pH 7.4). In the case of the loaded hybrid NPs, the release took place in 1 mL of the selected medium. The removal of the released PMX from the system at different time intervals was carried out concomitantly using an ultrafiltration method using membrane concentrators with a MWCO of 3000. After the first round of ultrafiltration, a corresponding aliquot of the medium was added to the NPs, and the solution was filtered again. The procedure was repeated three times. At each time point, the colloid was made up to 1 mL with the working medium. In the case of composite materials, the release took place in 500 µL 0.01 M PBS (pH 7.4). At the desired time points, the medium was changed to a fresh one. The collected solutions were combined, freeze-dried, and then analyzed with an Elute UPLC chromatograph (Bruker Daltonics GmbH, Bremen, Germany) connected with a Maxis Impact Q-TOF mass spectrometer (Bruker Daltonics GmbH, Bremen, Germany) equipped with an electrospray ionization (ESI) source (Bruker Daltonics GmbH, Bremen, Germany) using a previously published procedure [12].

#### 3.3.8. Antimicrobial Activity

The antimicrobial activity was studied by the microtiter broth dilution method described by the Clinical and Laboratory Standards Institute using *P. aeruginosa* (ATCC 27,853). The antimicrobial activity of PMX delivery systems based on the hybrid NP dispersions was assayed using a previously developed protocol [36].

For composite materials, the suppression of *P. aeruginosa* growth was assayed by the agar well diffusion method. The bacterial strain was cultured in Mueller–Hinton broth (HiMedia, Mumbai, India) at 37 °C. The OD of an overnight *P. aeruginosa* suspension in Mueller–Hinton broth was measured on a UV mini-1240 spectrophotometer (Shimadzu, Kyoto, Japan) at a wavelength of 540 nm and plated for enumeration of CFU on Luria-Bertani agar [53]. The culture suspension was then serially diluted in Mueller–Hinton broth to give approximately 1 × 10^7^ CFU/mL. A 20 mL volume of sterile Muller–Hinton Agar (Research Center for Pharmacotherapy [RICF], St. Petersburg, Russia) was poured into sterile Petri dishes (d = 100 mm). After solidification, 1 mL of inoculum at a concentration of 1 × 10^7^ CFU/mL was inoculated onto the Muller–Hinton Agar plates by rocking the Petri dish. Excess inoculum was removed with a pipette, and the Petri dishes were dried at room temperature (20–22 °C) for 10–15 min. Wells (6 mm diameter) were created in the agar using a sterile cylindrical template, 25 µL of colloids were added, and the wells were filled with nutrient medium. The amounts of PMXs loaded per well as a solution of free antibiotic or a dispersion of the delivery system were 37 ± 1 and 74 ± 2 µg per zone 1 and 2. In the case of hydrogels, circular pieces of hydrogel (6 mm diameter) were placed into the wells. Each hydrogel sample contained 85.6 ± 1.7 µg of PMX. The antimicrobial agents diffused to the nutrient medium and suppressed the growth of *P. aeruginosa*. The results were evaluated by measuring the diameter of the inhibition zones, including the diameter of the well.

## 4. Conclusions

Hybrid core–shell NPs, consisting of a silver core and poly(glutamic acid) shell, were developed as potential delivery systems for PMXs. The obtained hybrid NPs had a hydrodynamic diameter of approximately 100 nm and were capable of binding PMXs at amounts up to 450 µg per mg of NPs without aggregation. Moreover, the hybrid NPs did not show cytotoxicity at amounts up to 64 and 125 µg/mL for normal and cancer cells, respectively, and demonstrated quite low uptake by macrophages. Comparison of the developed PGlu@Ag with silver NPs stabilized by cysteine revealed that both types of NPs have similar biological properties. However, the PGlu-covered silver NPs are smaller, more stable, and allow PMX loading for combined antibacterial therapy. Approximation of the release data using several mathematical models has shown that the main release mechanism is diffusion, namely non-Fickian diffusion in the case of hybrid NPs and Fickian diffusion in the case of composite materials. The prepared PMX formulations based on the hybrid PGlu@Ag NPs demonstrated a MIC of 1 µg/mL against *P. aeruginosa*. Notably, the MIC was lower for the PMX B/PGlu@Ag delivery system than for the free antibiotic. This result can be attributed to the synergetic action of PMX and the silver NP core. The developed hybrid NPs showed antimicrobial properties when dispersed in a hydrogel. These composite hydrogels with combined antibacterial properties can be considered as potential candidates for the treatment of skin injuries, such as wounds and burns.

## Figures and Tables

**Table 1:** Characteristics of PGlu@Ag nanoparticles determined by dynamic and electrophoretic light scattering.

Conditions of NPs’ Redispersion	*D_H_* (nm)	PDI	ζ-Potential (mV)
H_2_O	97	0.44 ± 0.03	−57 ± 2
0.01 M PBS, pH 7.4	92	0.46 ± 0.02	−48 ± 5
0.02 M acetic buffer, pH 3.8	89	0.50 ± 0.01	−51 ± 3
0.02 M borate buffer, pH 10.5	88	0.50 ± 0.01	−55 ± 2

**Table 2:** Characteristics of Cys@Ag nanoparticles determined by dynamic and electrophoretic light scattering.

Conditions of NPs’ Redispersion	*D_H_* (nm)	PDI	ζ-Potential (mV)
H_2_O, 20 s	423	0.56 ± 0.01	−19 ± 1
0.01 M PBS, pH 7.4, 20 s	606	0.42 ± 0.03	−42 ± 3
0.02 M acetic buffer, pH 3.8, 45 s	505	0.32 ± 0.01	+39 ± 2
0.02 M borate buffer, pH 10.5, 30 s	780	0.36 ± 0.02	−53 ± 2

**Table 3:** Changes in characteristics of hybrid PGlu@Ag NPs after loading of polymyxin B.

Loaded PMX (µg/mg of NPs)	*D*_*H*_ (nm)	ζ-Potential (mV)
0	93 ± 54	−48 ± 2
330	184 ± 95	−41 ± 1
450	206 ± 107	−36 ± 1
830	1215 ± 675	−28 ± 2
1250	1945 ± 874	−10 ± 1

## Data Availability

Data available on request from the authors.

## Supplementary Materials

The following are available online at https://www.mdpi.com/article/10.3390/ijms23052771/s1.
