# Supplementary material for: Hybrid Nanoparticles and Composite Hydrogel Systems for Delivery of Peptide Antibiotics
Source: Int J Mol Sci. 2022 Mar 2;23(5):2771. doi: 10.3390/ijms23052771 (PMC8911036; doi:10.3390/ijms23052771)
Supplement: Supplementary file 1 [file ijms-23-02771-s001.zip › ijms-1600328-supplementary.pdf]

International Journal of
Molecular Sciences

MDPI

Supplementary Materials

Hybrid Nanoparticles and Composite Hydrogel Systems for Delivery of Peptide Antibiotics

Dmitrii Iudin [1], Marina Vasileva [1], Elena Knyazeva [2], Viktor Korzhikov-Vlakh [3], Elena Demyanova [2], Antonina Lavrentieva [4], Yury Skorik [1] and Evgenia Korzhikova-Vlakh [1]

1 Institute of Macromolecular Compounds, Russian Academy of Sciences, Bolshoi VO 31, 199004 St. Petersburg, Russia
2 State Research Institute of Highly Pure Biopreparations, Pudozhsakya 7, 197110 St. Petersburg, Russia
3 Institute of Chemistry, St. Petersburg State University, Universitetskii 26, Peterhof, 198504 St. Petersburg, Russia
4 Institute of Technical Chemistry, Gottfried-Wilhelm-Leibniz University of Hannover, 30167 Hannover, Germany

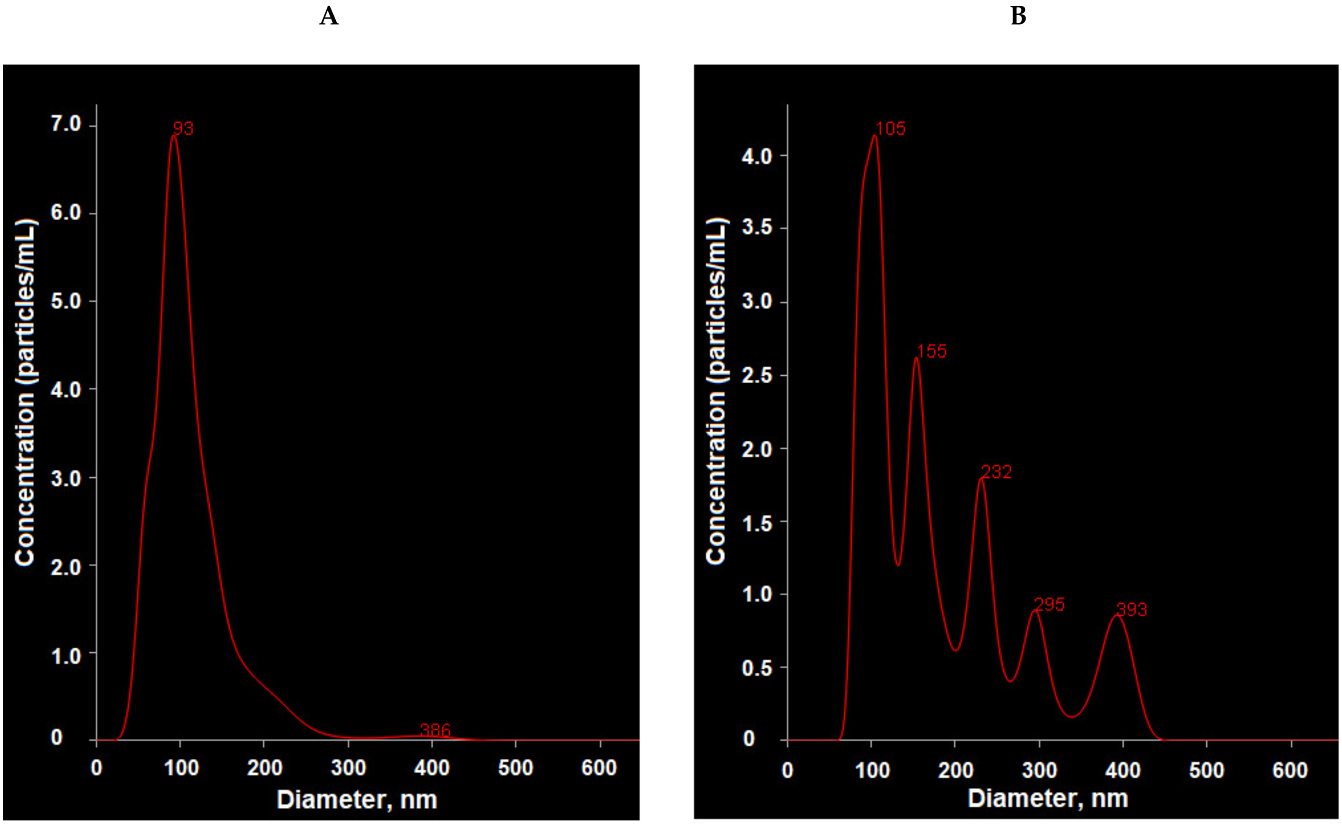

Figure S1. Nanoparticle tracking analysis (NTA) of PGlu@Ag NPs (**A**) and Cys@Ag NPs (**B**).

Table S1. Correlation coefficients and calculated parameters with different mathematical models of release.

**Model**	**Hybrid NPs**			**Gels**		
	PMX B		*PMX E*	*PMX B*		
	0.01 M PBS (pH 7.4)	Simulated Plasma	0.01 M PBS (pH 7.4)	Agarose in 0.01 M PBS (pH 7.4)	Agarose+Hybrid NPs in 0.01 M PBS (pH 7.4)	Agarose+Hybrid NPs in 0.01 M AcOH/AcONa (pH 5.5)
**Zero-order***	R^2 = 0.8323<br>K_{zo} = 0.43	R^2 = 0.8420<br>k_{zo} = 2.59	R^2 = 0.8546<br>k_{zo} = 0.74	R^2 = 0.6176<br>k_{zo} = 3.32	R^2 = 0.7103<br>k_{zo} = 2.21	R^2 = 0.6453<br>k_{zo} = 2.84
**First-order ***	R^2 = 0.8383<br>k_{fo} = 5.0×10^{-3}	R^2 = 0.8978<br>k_{fo} = 4.5×10^{-2}	R^2 = 0.8644<br>k_{fo} = 8.3×10^{-3}	R^2 = 0.8440<br>k_{fo} = 0.15	R^2 = 0.7583<br>k_{fo} = 0.04	R^2 = 0.7664<br>k_{fo} = 0.07
**Higuchi ***	R^2 = **0.9274**<br>K_H = 1.95	R^2 = **0.9348**<br>K_H = 11.72	R^2 = **0.9415**<br>K_H = 3.42	R^2 = 0.8184<br>K_H = 16.50	R^2 = 0.8801<br>K_H = 10.74	R^2 = 0.8436<br>K_H = 14.02
**Korsmeyer-Peppas ****	R^2 = **0.9524**<br>K_{KP} = 1.76<br>n = 0.67	R^2 = **0.9635**<br>K_{KP} = 11.07<br>n = 0.65	R^2 = **0.9624**<br>K_{KP} = 4.09<br>n = 0.51	R^2 = **0.9676**<br>K_{KP} = 31.48<br>n = 0.31	R^2 = **0.9992**<br>K_{KP} = 23.18<br>n = 0.19	R^2 = **0.9894**<br>K_{KP} = 27.03<br>n = 0.30
**Hixon-Crowell *****	R^2 = 0.7971<br>K_{HC} = 8.5×10^{-4}	R^2 = **0.9019**<br>K_{HC} = 8.5×10^{-3}	R^2 = 0.8691<br>K_{HC} = 1.6×10^{-3}	R^2 = 0.7463<br>K_{HC} = 0.03	R^2 = 0.7434<br>K_{HC} = 5.6×10^{-3}	R^2 = 0.6589<br>K_{HC} = 8.2×10^{-3}
**Hopfenberg *****	R^2 = 0.7994<br>K_{Hb} = 9.9×10^{-6}	R^2 = **0.9195**<br>K_{Hb} = 2.7×10^{-5}	R^2 = 0.8726<br>K_{Hb} = 2.1×10^{-5}	R^2 = 0.8459<br>K_{Hb} = 6.6×10^{-5}	R^2 = 0.7599<br>K_{Hb} = 7.9×10^{-5}	R^2 = 0.6976<br>K_{Hb} = 2.3×10^{-5}
**Baker-Lonsdale *****	R^2 = **0.9014**<br>K_{BL} = 4.4×10^{-5}	R^2 = **0.9599**<br>K_{BL} = 2.5×10^{-3}	R^2 = **0.9478**<br>K_{BL} = 1.5×10^{-4}	R^2 = 0.8134<br>K_{BL} = 4.8×10^{-3}	R^2 = 0.8843<br>K_{BL} = 1.6×10^{-3}	R^2 = 0.8267<br>K_{BL} = 2.9×10^{-3}
**Weibull ******	R^2 = **0.9954**<br>α = 22.85<br>β = 0.20	R^2 = **0.9937**<br>α = 5.62<br>β = 0.48	R^2 = **0.9961**<br>α = 16.40<br>β = 0.30	R^2 = **0.9983**<br>α = 1.52<br>β = 0.10	R^2 = **0.9991**<br>α = 3.54<br>β = 0.19	R^2 = **0.9959**<br>α = 2.05<br>β = 0.11
**Gompertz ******	R^2 = **0.9598**<br>α = 3.66<br>β = 0.24	R^2 = **0.9833**<br>α = 2.46<br>β = 1.12	R^2 = **0.9915**<br>α = 3.12<br>β = 0.35	R^2 = **0.9582**<br>α = 1.05<br>β = 0.49	R^2 = **0.9993**<br>α = 1.48<br>β = 0.36	R^2 = **0.9773**<br>α = 1.20<br>β = 0.40
**Peppas-Sahlin ******	R^2 = **0.9667**<br>K_1 = 2.64<br>K_2 = 0.17<br>m = 0.44	R^2 = **0.9887**<br>K_1 = 14.73<br>K_2 = 0.53<br>m = 0.44	R^2 = **0.9882**<br>K_1 = 5.02<br>K_2 = 0.23<br>m = 0.38	R^2 = **0.9701**<br>K_1 = 40.18<br>K_2 = 6.36<br>m = 0.33	R^2 = **0.9977**<br>K_1 = 26.51<br>K_2 = 3.58<br>m = 0.25	R^2 = **0.9867**<br>K_1 = 34.13<br>K_2 = 5.35<br>m = 0.32

Zero-order $F=k_{zo}*t$

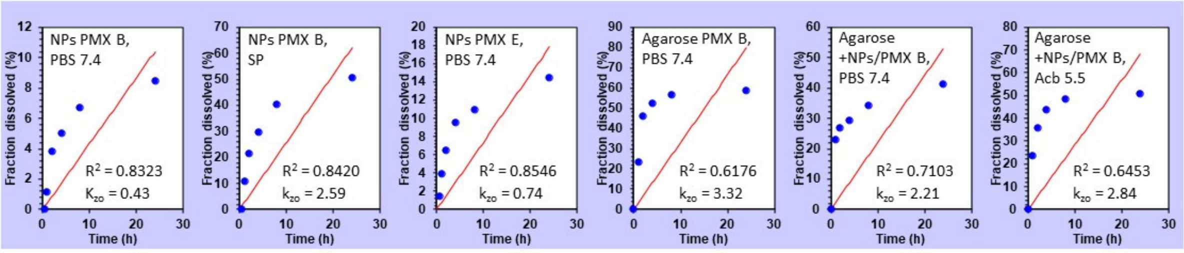

First-order $F=100*[1-Exp(-k_{fo}*t)]$

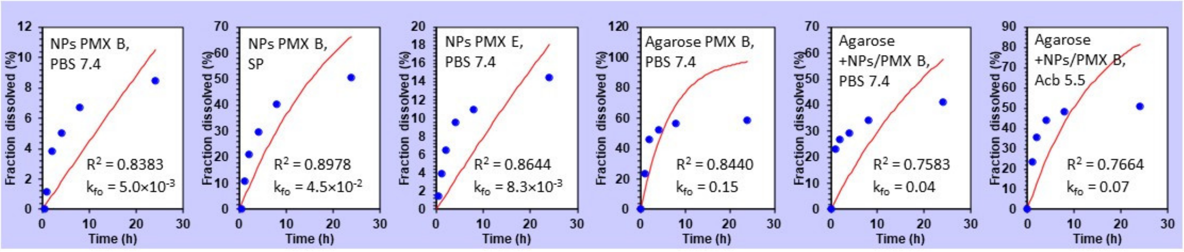

Higuchi $F=k_H*t^{0.5}$

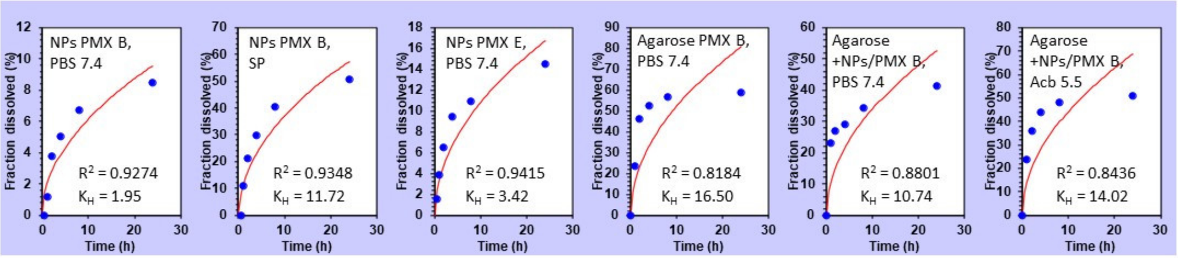

Korsmeyer-Peppas $F=k_{KP}*t^n$

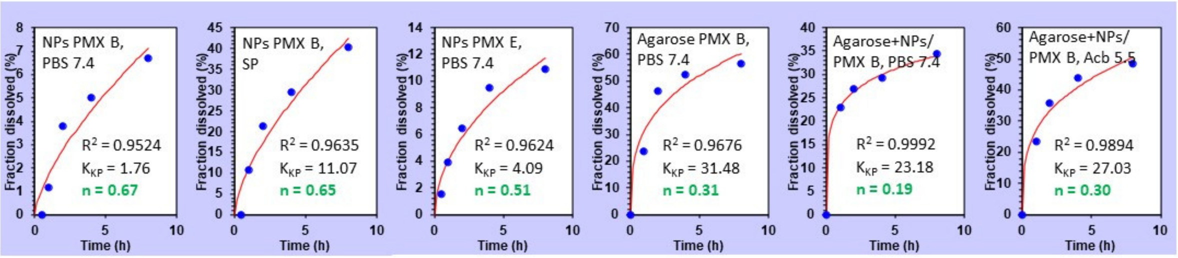

Hixson-Crowell $F=100*[1-(1-k_{HC}*t)^3]$

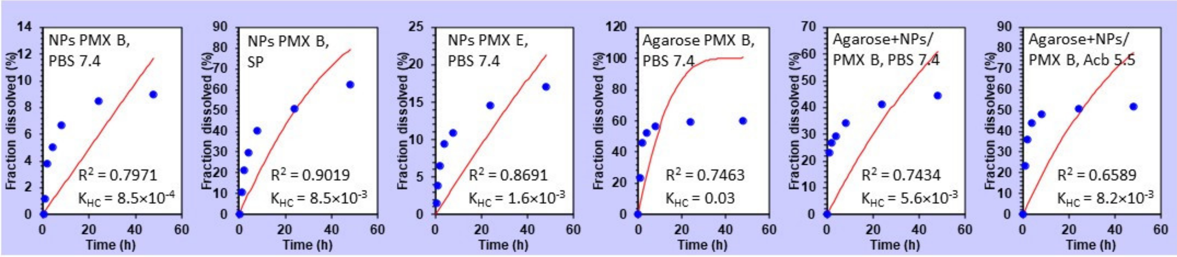

Hopfenberg $F=100*[1-(1-k_{HB}*t)^n]$

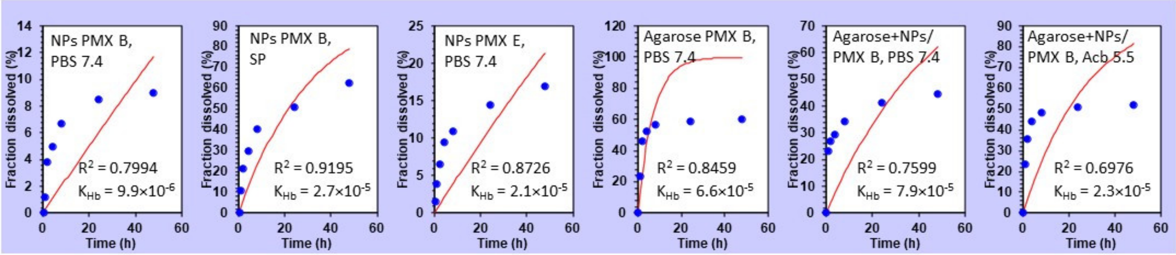

Baker-Lonsdale $3/2*[1-(1-F/100)^{(2/3)}]-F/100=k_{BL}*t$

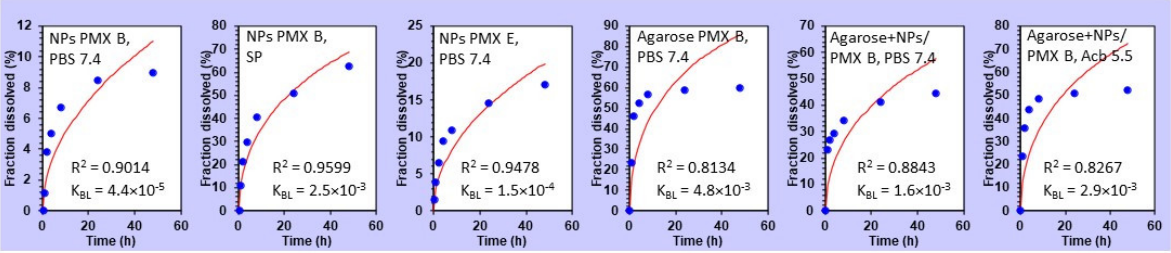

Weibull $F=100*\{1-Exp[-(t-Ti)^\beta/\alpha]\}$

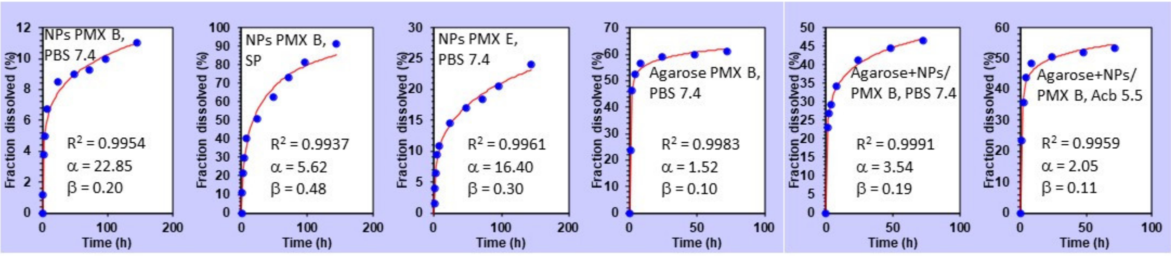

Gompertz $F=100*Exp\{-\alpha*Exp[-\beta*log(t)]\}$

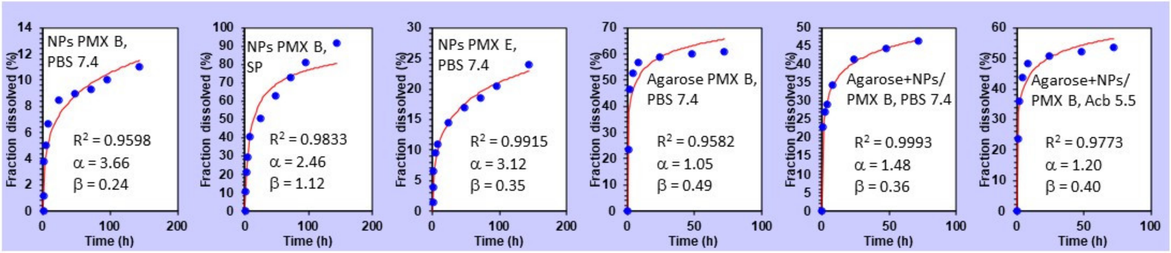

Peppas-Sahlin $F=K_1*t^m+K_2*t^{(2*m)}$

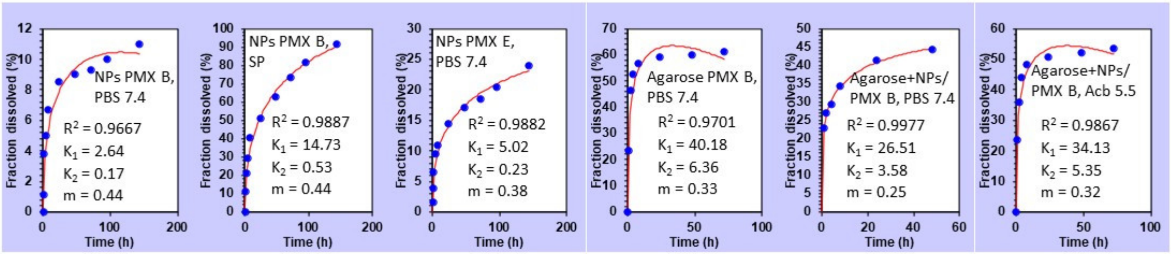

Figures S2. Regression curves, correlation coefficients and calculated parameters for different mathematical models of release.
